# Solution-Based Research to Address Disparities in Precision Cancer Health: A Case Report Elucidating the Design and Rationale of the Illinois Cancer Health Equity Research (I-CHER) Center

**DOI:** 10.3390/ijerph23040446

**Published:** 2026-03-31

**Authors:** Frank A. Granata, Aileen Shen, Karissa Cerda, Monet Jones, Osei Bekoe, Erica Seltzer, Julie Bobitt, Margaret Wright, Paola Torres, Carolina Bujanda, Angelina Izguerra, L. A. Naiche, Vivian Pan, Ana Waite, Keith Naylor, Patrick Smith, Ryan Nguyen, Leslie Carnahan, Vida Henderson, Kent Hoskins, Chinwe Ewenighi, Hunter K. Holt, Shaveta Khosla, M. J. Godoy-Calderon, Saria Lofton, Ines Pulido, Ameen Salahudeen, Elizabeth Rivera, Joanne Glenn, Candace Henley, Charles Walton, Karen Sharer, Ally Lopshire, Marcus Evans, Ian Jasenof, Vijayakrishna Gadi, Pamela Ganschow, Jan Kitajewski, Marian Fitzgibbon, Yamilé Molina

**Affiliations:** 1 University of Illinois Cancer Center, University of Illinois College of Medicine, 818 S Wolcott Ave, Chicago, IL 60612, USAacshen@uic.edu (A.S.); kcerda2@uic.edu (K.C.); mnjones1@uic.edu (M.J.); oseib@uic.edu (O.B.); eseltzer@uic.edu (E.S.); mewright@uic.edu (M.W.); ptorres4@uic.edu (P.T.); carolabu@uic.edu (C.B.); adi5@uic.edu (A.I.); vivianp@uic.edu (V.P.); awilli97@uic.edu (A.W.); knaylor@uic.edu (K.N.); psmith26@uic.edu (P.S.); rnguye8@uic.edu (R.N.); lcarna2@uic.edu (L.C.); vhende5@uic.edu (V.H.); khoski@uic.edu (K.H.); chinwe2@uic.edu (C.E.); hholt2@uic.edu (H.K.H.); skhosl2@uic.edu (S.K.); mjgodoy@uic.edu (M.J.G.-C.); slofto4@uic.edu (S.L.); ipulid3@uic.edu (I.P.); ameen@uic.edu (A.S.); elizabeth.riverawin62@gmail.com (E.R.); cewalton2@gmail.com (C.W.); jasenof@uic.edu (I.J.); vkgadi@uic.edu (V.G.); pgansch@uic.edu (P.G.); kitaj@uic.edu (J.K.); mlf@uic.edu (M.F.); 2 Division of Community Health Sciences, University of Illinois Chicago School of Public Health, 1603 W. Taylor St., Chicago, IL 60612, USA; 3 Department of Medicine, University of Illinois College of Medicine, 840 South Wood Street, Suite 1020N, MC 787, Chicago, IL 60612, USA; jbobitt@uic.edu; 4 Department of Physiology and Biophysics, University of Illinois College of Medicine, 835 S Wolcott Ave, E-202, Chicago, IL 60612, USA; naiche@uic.edu; 5 Mile Square Health Center (FQHC), 1220 S Wood Street, Chicago, IL 60608, USA; 6 Department of Population Oral Health, University of Illinois College of Dentistry, 801 S. Paulina (MC 621), Chicago, IL 60612, USA; 7 Department of Family and Community Medicine, University of Illinois College of Medicine, 1919 West Taylor Street, Room 196 Chicago, IL 60612, USA; 8 Department of Population Health Nursing Science, University of Illinois College of Nursing, 845 S. Damen Ave., MC 802, Chicago, IL 60612, USA; 9 Department of Surgery, University of Illinois College of Medicine, 840 S Wood St., Chicago, IL 60612, USA; 10 AI.Health4all Center for Health Equity, University of Illinois College of Medicine, 1853 W Polk St., Chicago, IL 60612, USA; 11 Department of Biochemistry and Molecular Genetics, University of Illinois College of Medicine, 900 S Ashland Ave, Chicago, IL 60607, USA; 12 Women on Top of Their Game, 46 East 26th Street, Chicago, IL 60616, USA; doctorjaygee@gmail.com; 13 The Blue Hat Foundation, 46 E 26th St., Chicago, IL 60616, USA; candace@bluehatbowtie.org; 14 American Cancer Society, 150 S Wacker Dr. Suite #2100, Chicago, IL 60606, USA; karen.sharer@cancer.org; 15 American Cancer Society Cancer Action Network, 655 15th Street, NW, Suite 503, Washington, DC 20005, USA; ally.lopshire@cancer.org; 16 State Rep. Marcus C. Evans, Jr.—33rd District, 8500 S Stony Island Ave, Chicago, IL 60617, USA; marcus@repevans.com; 17 Department of Pediatrics, University of Illinois College of Medicine, 840 S Wood St., Chicago, IL 60612, USA

**Keywords:** cancer, health disparities, community engagement, community-clinical-academic partnerships

## Abstract

**Highlights:**

**Public health relevance—How does this work relate to a public health issue?**

- The work conducted by the I-CHER Center directly addresses persistent, multi-faceted cancer health disparities and highlights the multilevel factors that drive them—including structural, social, and biological determinants—which result in a disproportionate burden of advanced-stage cancer diagnosis and worse outcomes for highly vulnerable populations.
- The Center’s mission centers around addressing the critical public health issue of inequitable access to precision cancer health, with a particular focus on the barriers low-income and minoritized communities face in obtaining risk assessment, reduction, and appropriate cancer care.

**Public health significance—Why is this work of significance to public health?**

- This case report provides a reproducible model for establishing a multi-sectoral administrative infrastructure within a Minority Serving Institution (MSI) and a Federally Qualified Health Center (FQHC) to advance scientific solutions in precision cancer health equity.
- The Center’s approach has demonstrated significant population-level reach, cumulatively engaging over 60 healthcare providers in precision cancer health strategies and serving over 10,000 residents from under-resourced communities.

**Public health implications—What are the key implications or messages for practitioners, policy makers and/or researchers in public health?**

- I-CHER Center initiatives have demonstrated how integrating policy advocates and legislative champions into the research structure can offer a powerful mechanism to achieve rapid translation of evidence into state law that can comprehensively address systemic barriers like the financial cost of genetic testing and lack of clinical trial diversity.
- The Center elucidates how achieving sustainable cancer health equity requires robust, long-term community-academic-systems collaborations and partnerships to successfully investigate, implement, and sustain precision cancer health strategies within FQHCs and other safety-net clinical environments.

**Abstract:**

Equity in precision cancer health, defined here as equitable access to inclusive cancer risk assessment, cancer risk reduction/management, and risk-appropriate cancer healthcare from prevention through survivorship, is critical for addressing broader population cancer disparities. Specifically, we describe the impact of the Illinois Cancer Health Equity Research (I-CHER) Center on precision cancer health equity, including how the Center cumulatively served >10,000 residents from under-resourced communities; disseminated findings to >300 members of the local cancer health disparities workforce; and translated scientific solutions into sustained clinical practice and two state laws. The objective of this case report is to describe the I-CHER Center’s multisectoral structure; participatory administrative processes for research; early implementation challenges and tensions across sectors; and solutions that contributed to early center-wide successes. This case report offers one example of the administrative infrastructure needed for advancing scientific solutions in precision cancer health equity within a Minority Serving Institution (MSI) and its internal federally qualified health center (FQHC).

## 1. Introduction

Stark, multi-faceted cancer health disparities persist across cancer types, minoritized populations, and the care continuum, despite significant reductions in overall cancer mortality [1,2,3,4,5,6,7,8,9,10,11,12,13,14,15,16,17,18]. Biological, social, and structural factors drive these disparities through independent and intersectional pathways [19,20,21,22,23,24,25,26,27]. Individuals, families, and communities with elevated biological, social, and structural cancer risks bear the disproportionate burden of population-level cancer health disparities [28,29,30,31,32,33,34,35]. Decades of transdisciplinary research have advanced conceptualization and knowledge on concurrent, independent roles that biological, social, and structural factors contribute to cancer health disparities [19,29,30,31,32,33,34,36].

Research has highlighted the interactive roles of multilevel determinants, including through equity in precision cancer health. Here, we define equity in precision cancer health as inclusive cancer risk assessment, cancer risk reduction/management, and risk-appropriate cancer healthcare from prevention through survivorship. Equity in precision cancer health is critical, given increasingly personalized guidelines across the cancer care continuum and the growing use of precision oncology to address the complex etiology and clinical outcomes that cancer represents. However, low-income communities are less likely to participate in the first step of precision cancer health—cancer risk assessment (e.g., documentation of family history, tobacco pack years; genetic and biomarker testing)—than high-income communities [3,5,8,11,14,15]. Simultaneously, disparities in cancer incidence partially reflect social stratification, which catalyzes lifestyle cancer risks (e.g., 18–44% higher adult obesity rates in 2011–2022) [37], and a lack of access to appropriate healthcare for biological risks (e.g., family history, pathogenic variants). Finally, low-income survivors are unable to benefit from risk-informed survivorship guidelines [38,39,40], due to less participation in cancer risk assessment and tailored risk reduction programs (e.g., cardiovascular complications) [1,2,9,13,16,17,18].

Cook County exemplifies national disparities in access to precision cancer health [41,42]. For example, residents of federally designated medically underserved areas/populations (MUA/Ps) in Cook County suffer 225% more transportation challenges, 196% greater violent crime, 100% greater need to use food stamps, and 40% more housing instability than the nation [43,44,45,46]. These social and structural risks catalyze an alarming surge of cancer risks for MUA/P residents, who face 12% greater obesity, 17% less exercise, and 7% more smoking relative to the US overall [37,47]. Simultaneously, healthcare systems embedded in MUA/Ps have limited access to cutting-edge tools for precision cancer health, from cancer risk assessment through personalized cancer survivorship care [48]. The lack of access to precision cancer healthcare—coupled with disturbingly high lifestyle risks—are estimated to contribute to 38–133% greater rates of early-onset breast and prostate cancers diagnosed at an advanced stage, 17–28% greater incidence rates of lung and colorectal cancers, and 17–27% greater late stage diagnosis across these cancers for MUA/Ps relative to the national average [49,50]. This multidimensionally limited access to precision cancer health drives late-stage diagnosis and worse outcomes among the most vulnerable members of low-income communities [4,6,10,12].

There is an urgent need for basic, translational, and population sciences research that addresses both elevated biological risks (e.g., pathogenic variants) as well as elevated social and structural risks (e.g., lack of insurance, residence in persistently impoverished neighborhoods). Successful strategies must be effectively and nimbly translated to practice and policy, to claim the full promise of precision cancer health for everyone. Several research centers have been successfully launched to advance cancer health equity, leading to early blueprints for administrative infrastructure for cancer health equity research overall [51,52,53,54,55,56]. Past centers have largely, albeit not exclusively, been operated by NCI-designated cancer centers and other well-resourced academic partners. Such academic partners nourish and advocate for communities, but are not usually part of the safety net ecosystem (e.g., limited Medicaid coverage, charity care). Furthermore, their partnerships with communities may differ from the resource constraints, structures, and experiences of safety net healthcare systems and commuter/public minority-serving institutions (MSI) that are physically embedded in MUA/Ps.

The current case report offers one example of how to establish an administrative infrastructure for precision cancer health equity within the setting of an MSI and its internal federally qualified health center (FQHC). The Illinois Cancer Health Equity Research (I-CHER) Center focuses on advancing the broadest access to the highest quality precision cancer health, through the Cancer Health Research Center (CHERC) program sponsored by the American Cancer Society (ACS). The objective of this case report is to describe the I-CHER structure, administrative processes, early challenges, and early successes in research, practice, and policy efforts.

## 2. Detailed Case Description

We first describe the setting of the I-CHER Center, with a focus on how a public MSI and its safety net medical center were able to launch a large-scale initiative on precision cancer health equity. Next, we describe the center’s multi-sectoral structure, processes, early challenges and tensions, solutions, and early outcomes for each I-CHER Center priority (1—research reach, 2—research translation to practice and policy). This case report focuses on the Center, as I-CHER study teams have previously published their study designs and lessons learned [57,58,59,60,61,62].

### 2.1. Setting

Table 1 depicts the overall structure of the I-CHER Center, which was led by a multi-sectoral Administrative Core (AC) that comprised academic, clinical, and community leaders with equal decision power. An Investigator Development Core (IDC) supported the development of early-career faculty, with a focus on growing community-engaged scientists embedded in MSI and safety-net healthcare settings. Finally, the I-CHER Center’s Cores and research projects were shaped by a community advisory board (CAB) representing the multiple levels of influence that the I-CHER Center addressed (patient, community, healthcare systems, policy advocacy). Cores and the CAB roles across I-CHER research and research translation are detailed in Section 2.2 and Section 2.3. Below, we describe the institutional settings and capacity that nourished the I-CHER Center.

The University of Illinois Chicago (UIC) is a research-intensive, public MSI with a safety net medical center (UI Health) that includes a federally qualified health center (FQHC; Mile Square Health Center—Mile Square) and affordable specialty services (oncology, dentistry). UI Health and Mile Square sites are physically embedded in MUA/Ps across Cook County, with the goal of offering the highest quality clinical care to the broadest range of individuals [63,64,65,66]. UI Health and Mile Square exhibited high clinical capacity for equitable delivery of precision cancer health at the inception of the I-CHER Center in 2021, including system-wide cancer genetic services (e.g., hereditary cancer risk assessment, genetics counseling, genetic testing), cancer survivorship services, cancer risk reduction services (e.g., tobacco cessation/streamlined navigation to state quitlines), and cancer screening services (e.g., site for CDC breast and cervical cancer screenings) in its Mile Square FQHC settings [67,68,69,70]. Primary care capacity was augmented by streamlined clinical coordination pathways to cancer diagnostic and treatment services, through internal specialty services partners that shared the same electronic health record system, clinicians, and staff within UIC.

The I-CHER Center further leveraged a robust baseline infrastructure for community-clinical-academic partnerships through the University of Illinois Cancer Center (UICC). The UICC Community Outreach and Engagement (COE) team administratively led the I-CHER Center under the auspices of the UICC Director and I-CHER Center PI.

In line with NCI-designated and other emerging cancer centers, the UICC COE team’s institutional roles are to identify community priorities for science, facilitate community–clinical–academic partnerships in equity-driven cancer research, and enable translation to practice and policy. A key tenet for the COE-driven I-CHER Center was that multi-sectoral partnerships should drive multilevel scientific solutions in cancer health disparities, from discovery through sustainable implementation. The Center’s framework thus integrated well-established partnership models, including the Community–Campus Partnerships for Health and the Expanded Chronic Care Model, with robust socio-ecological cancer health disparities models [71,72,73]. As demonstrated below, I-CHER structure and processes were organized to enable: (1) mutual learning, benefits, and resource sharing; (2) trust and relationship building toward transparent, respectful, and relevant partnerships in research on precision cancer health and multilevel drivers; (3) shared experiences in decision-making and ownership across the Center and its components; and (4) agency toward action, toward expedited translation to public health practice and policy [74,75,76].

### 2.2. Advancing Precision Cancer Health Equity Research

The I-CHER Center ultimately funded 12 projects (Table 2), including Research Scholar Grants (RSGs), which are R01-equivalent studies; Clinical Scientist Development Grants (CSDGs); postdoctoral fellowships (PF); and pilot project grants. Project duration varied, with larger-scale initiatives lasting 3–4 years (RSGs, CSDGs, PFs) and pilot initiatives lasting one year. Below, we describe how research projects were solicited, reviewed, and monitored through participatory processes within the Center’s Cores and CAB.

#### 2.2.1. Multi-Sectoral Infrastructure for Soliciting, Reviewing, and Selecting Projects

The Center Cores were charged with overseeing project solicitation, review, and selection for the I-CHER precision cancer health research portfolio. Key criteria that were promoted, when soliciting grants, and used for project evaluation included: (1) relevance in addressing catchment priorities (e.g., priority cancers—breast, colorectal, prostate, lung, liver) through a precision cancer health lens (i.e., genetic and structural/social risks), based on UICC COE longitudinal cancer health needs assessments and augmentation with representative population data; (2) the relevance in addressing clinical priorities (e.g., Mile Square FQHC—Uniform Data System metrics; UI Health oncology—Commission on Cancer standards) and leveraging past clinical investments/quality improvement initiatives in precision cancer health for UI Health and Mile Square (see above); and (3) strong representation on the study teams that could facilitate intervention across proposed levels of influence. For example, study teams focusing on patient- and community-level interventions ideally incorporate patient and community co-investigators/consultants. Study teams proposing provider and healthcare system interventions ideally comprised clinicians, faculty, and leaders from the “target” clinical units (e.g., emergency department, dentistry).

The I-CHER Center used multi-tiered processes for engaging diverse reviewers in research solicitation, review, and selection. As strategically planned by the I-CHER PI/UICC Director, these I-CHER processes were overseen by COE, given the team’s institutional obligations to monitor catchment priorities for informing science and their roles as “brokers” to enable multi-sectoral decisions about community-driven scientific investments. For initial projects (3 RSGs, 2 CSDGs, 1 PF), the AC—including UICC, UI Health/Mile Square, and CAB leaders—oversaw project solicitation, review, and selection. The AC convened multiple times to establish group consensus, review the key criteria above, and vote, using a majority rule. After the Center was funded, the IDC oversaw the pilot program, which facilitated the solicitation, review, and awarding of 1-year pilot projects led by early-career scientists. Request For Applications (RFA) were widely distributed to early-stage investigators through UICC listservs and the Cancer Research Education and Training Coordination (CRTEC) office. Grant reviewers included senior UICC researchers, UI Health leadership, and CAB members. To ensure diverse perspectives, grant reviewer opportunities were also offered to COE partners at large and I-CHER investigators (e.g., CSDG PIs, previous pilot project awardees). Grant reviewers for pilot projects underwent brief training of the I-CHER Center that included key criteria described above and its goals, and engaged with IDC leadership facilitators. Initial recommendations were shared with the I-CHER AC, including the UICC Director, CAB leaders, and UI Health leaders. The AC made the ultimate funding decisions using a voting majority rule. Awarded applications were largely selected because they represented testable, solution-based projects that focused on equity in precision cancer health, in terms of addressing biological and social determinants (e.g., translational research for cost-effective, targeted immunotherapy and survivorship) and were feasible (e.g., reliance on existing electronic medical record [EMR] data).

Reported challenges for initial project review and selection included the need for training about what I-CHER prioritized and the need for more structured instruments for grant review, given the diverse perspectives represented in “study sections.” This feedback directly contributed to the pilot project program infrastructure described above, including structured training and evaluation forms.

Other early tensions emerged during the launch of the pilot program, including (1) logistic concerns about “study section” discussions; and (2) patient and community reviewers’ concerns about the misalignment of grant applications with community priorities and the I-CHER Center’s focus on solution-based research in precision cancer health. Community partners with expertise in scientific grant review advocated that I-CHER processes be changed to parallel processes that national funders operationalize (e.g., Department of Defense, ACS). Specifically, non-academic reviewers preferred first hearing their academic counterparts’ perspectives on the studies, especially for basic and translational studies, and emphasized their review primarily on the fit with community priorities and clinical feasibility. Subsequent cycles of review aligned with these recommendations, in terms of having academic partners share their reviews and feedback first and having community partners as equal reviewers who were primarily engaged in whether the research fit community priorities. Structured forms for grant review also facilitated community and clinical reviewers’ evaluation of potential translation to practice and policy, if successful. Other concerns, led by patients and community reviewers, regarded the limited applications on cancer survivorship, for targeted risk reduction, and intervention development/testing. Another concern regarding study team representation is that pilot project applicants were often early-career investigators with limited collaboration networks. The AC solicited feedback from reviewers, including these concerns, annually. Group discussions across UICC, UI Health/Mile Square, and CAB leaders, led to different solutions, including (1) greater emphasis on cancer survivorship and intervention development for RFA promotional materials in subsequent cycles; and (2) information for level-setting among grant reviewers, regarding time constraints of projects (only one year) and the priority applicants for projects (i.e., early career investigators).

#### 2.2.2. Multi-Sectoral Infrastructure for Monitoring and Supporting Awarded I-CHER Research

Overall, the AC was charged with monitoring and supporting awarded I-CHER projects, with the IDC providing additional career development support, and the CAB providing feedback during quarterly CAB meetings and yearly I-CHER forums.

Monitoring and supporting awarded projects included; (1) development, distribution, and evaluation of structured progress reports for tracking milestone achievement and interim progress during AC meetings; (2) staffing support for study coordination and implementation; (3) support for regulatory processes, including approval from the Mile Square Research Council (for FQHC-based studies only), UICC Protocol Review Committee, and UIC institutional review board; (4) navigating I-CHER teams to UICC resources for common implementation risks (e.g., recruitment, intervention delivery, partnership dynamics); and (5) support for measurement and evaluation, including through UICC Shared Resources. Given limited funding, the UICC PI facilitated cost-sharing, to ensure I-CHER study teams had no-cost access to UICC Admin Core resources, including senior regulatory specialists, for coordinating approvals across the different institutional committees; the UICC Clinical Trials Office, COE, and Shared Resources for identifying eligible patients/partners, culturally astute recruitment and retention, and partnership management; and, Biostatistics Shared Resources Core and Data Integration Shared Resources Core for data abstraction, measurement and evaluation. Additionally, the UICC PI further approved cost-sharing for staff necessary for I-CHER projects, especially CSDGs. Through quarterly progress reports and yearly presentations by the study team, AC tracked study teams’ needs, referrals to UICC/institutional resources, and uptake of recommended resources.

To note, the purposive selection of study teams that were embedded in priority communities and clinical settings reduced common implementation risks and challenges. For example, several study PIs were clinicians, faculty, or leaders in the UI Health clinical units wherein their I-CHER research was conducted. System and provider interventions were thus facilitated, given that study PIs were part of the departmental network and/or because projects were framed in line with non-research departmental activities and standard care. Relatedly, study teams focused on community interventions leveraged robust partnerships with community consultants and investigators through the UICC COE office, which impacted the evaluation of new I-CHER projects.

Nonetheless, common implementation challenges persisted, especially for pilot projects. The COE-fueled AC and CAB supported recruitment challenges for patients and community partners, including advising on recruitment procedures (e.g., effective ‘cold calls’) and offering alternative recruitment sources (e.g., community recruitment, in addition to FQHC recruitment). For several pilot projects, facilitated by UICC COE and the AC, CAB members proactively joined study teams, with the goal of leveraging innovative interventions (e.g., Food is Medicine) for their constituents.

There were also challenges in implementation in safety net/FQHC settings—even when clinicians led interventions, if projects were “high touch” and required extensive time commitments (e.g., for trainings, clinical workflows). To address these challenges, the UICC Director and UI Health/Mile Square leadership on the AC identified several venues for improving buy-in and implementation, including: (1) structured consultations with the Mile Square Research Program, for identifying low-cost adaptations to study recruitment and intervention implementation; (2) monthly review of I-CHER projects, as a whole, with the Mile Square Quality Improvement Program, for re-framing studies and embedding them in evolving quality improvement initiatives; and (3) facilitating engagement with providers and leadership through established departmental meetings (e.g., All Staff Meetings, All Provider Meetings) to increase awareness of projects, disseminate results, and offer time for brief remarks. Overall, these additional administrative requests were facilitated by the leadership roles of I-CHER AC members within UI Health/Mile Square, who had standing institutional meetings and roles wherein I-CHER project feedback and strategies could be integrated.

#### 2.2.3. Initial I-CHER Research Reach

The I-CHER administrative infrastructure and processes described above have contributed to early successes in expanding access to precision cancer health equity across system, provider, and patient levels. Simultaneously, methodological innovations in basic and translational science have been framed and considered in terms of future applications for precision cancer health equity. Overall, the I-CHER research portfolio served >10,000 unique Cook County MUA/P residents through precision cancer health equity interventions and studies at multiple levels of influence. Below, we provide an early qualitative synthesis of “high tech” and “high touch” strategies across different levels of influence.

At the FQHC/safety net systems level, I-CHER strategies were launched across Mile Square FQHC clinics as well as oncology and emergency medicine departments within UI Health, by PIs who were clinicians and research faculty within those departments. “High tech” strategies included, for example, After Visit Summaries (AVS) for emergency medicine patients that identified the specific cancer screenings patients needed to obtain, based on individual risks and past care utilization data in the EMR. Examples of “high touch” were different clinic-level workflows among care teams and coordination strategies between care teams and community health workers (CHW) from partnering organizations. Beyond I-CHER’s institutional context, solution-based research also leveraged established collaboratives representing UIC and multiple healthcare systems (e.g., CAPriCORN), to understand and propose inter-facility care coordination interventions.

At the provider level, I-CHER research engaged providers for advising on system-level strategies and training them on provider-level strategies. Provider-level strategies included provider education modules embedded in EMR systems and communication toolkits for risk identification and screening recommendations. Given I-CHER’s goal of informing system-level strategies at UI Health and beyond, research teams solicited input from and trained 64 providers across primary care (Mile Square FQHC, internal medicine, family medicine), emergency medicine, dental, and oncology departments at UI Health and six external community clinics and safety net oncology departments. Study teams engaged in system strategies and provider education were often peers within the departments they engaged; as clinicians in primary care and specialty services, they sought to adapt.

At the patient level, I-CHER research engaged patients for their perspectives on intervention development and for intervention delivery services (*n* = 78). Patient-level strategies included culturally tailored educational videos that could be viewed in clinic and community settings, as well as health education sessions across phone and in-person formats. Research teams focused on patient interventions (1) comprised survivors who were community advocates with a long track record of health promotion in their communities and research partnerships with UICC researchers; and (2) included academic partners who embedded individual interventions within multilevel interventions and frameworks, to recognize patients as critical partners in their own care processes and simultaneously ensure supportive contexts for personal behavior change and shared decision making.

I-CHER’s research into biological risk factors was led by early-career faculty and postdoctoral trainees invested in molecular drivers of cancer disparities and, specifically, fundamental science to ensure the widest access to the most inclusive cancer medicine and treatment of the future (Table 2). I-CHER’s equity-driven approach is unique in its application of fundamental research methods—including various mouse models (e.g., ACKR1 knock-outs), cell lines (e.g., 4T1 cells, drug-resistant to MRTX1133), patient biospecimens, and new screening technologies (e.g., circulating fragmentomics)—to MUA/P, a demographic sparsely represented in such mechanistic studies. These studies were focused on solutions that could be widely accessible in terms of delivery in primary care/FQHC settings (e.g., blood draws for lung cancer screenings) and community oncology settings (e.g., point-of-care therapies).

### 2.3. Translating Precision Cancer Health Equity Research to Practice and Policy

Early research dissemination and translation successes for the I-CHER Center include dissemination of research findings to >300 members of the local cancer health disparities workforce; translation of three I-CHER interventions so far into sustained clinical practice at UI Health/Mile Square; and data-driven advocacy that contributed to two state laws for precision cancer health equity. Research translation processes were driven largely by the AC, in collaboration with the CAB, as described below.

#### 2.3.1. Research Dissemination

The I-CHER Center AC disseminated research findings through various institutional channels, including a regularly updated website; annual I-CHER forums for dissemination updates; research dissemination through UICC COE’s radio shows, which >10,000 African American and Latine residents in Cook County, every month; and invitations to present I-CHER findings to local, state, and national coalitions and convenings of the community health and healthcare service workforces (e.g., Alianza Americas, Illinois Community Health Worker Association; ACS Impact Makers in Chicago, 100 Black Men Chicago’s Annual Expo), as facilitated by UICC COE and the I-CHER CAB. Altogether, I-CHER teams have reached >300 community health workers and primary care teams while disseminating I-CHER research.

Challenges in research dissemination included (1) limited reach of intended audiences; and (2) timing between research implementation and translation, to ensure “live updates” at departmental meetings would not impact ongoing system- and provider-level interventions. The limited reach of dissemination channels largely pertained to the I-CHER website and annual forums, especially for safety net/FQHC providers, teams, and leaders, due to competing priorities. Certain solutions crafted by the AC included sharing findings through already established departmental meetings, as discussed above. Simultaneously, I-CHER study teams were concerned about the dissemination of active projects, especially within Cook County, because of potential contamination and impacts on end-users (e.g., patients, providers). To address this concern, the I-CHER AC organized research dissemination, such that the AC would first engage with study teams’ presentations and advise on how to share exciting information for continued buy-in across system, provider, and patient levels (e.g., accrual rates; summary statistics, without sharing outcome differences by study arm).

#### 2.3.2. Research Translation to Institutional Practice

At least three I-CHER interventions were sustained in standard care and practice within our institution, due to multiple successful processes. First, clinical leaders were incorporated into the I-CHER network (e.g., Mile Square Chief Medical Officer on I-CHER CAB) and were engaged through regular meetings and data collection efforts. Dissemination discussed during departmental meetings, as described above, further facilitated departmental buy-in and perceived co-ownership of interventions. Second, “high tech” strategies were developed that have low or no cost to maintain within existing clinical infrastructure—making them easy to adopt for clinical departments. Third, piloted “high touch” strategies, such as clinical workflows, were initially selected, as they required significant initial investment but relatively modest maintenance costs. Finally, conversations with clinical leaders and their units incorporated rich data on the effectiveness, feasibility, and acceptability of different strategies.

With regard to the specific sustained interventions, UI Health will sustain (1) modified AVS to promote risk-based cancer screening recommendations among emergency medicine patients, (2) use of the video tool for promoting shared decision making among Black breast cancer patients, and (3) use of electronic hereditary cancer risk assessment tool for collection of family history information and assessment of eligibility for cancer genetic services. For “high touch” strategies, Mile Square is considering the different clinic-level approaches to streamline genetic testing referrals by incorporating medical assistants (MAs), maintaining EMR tools (e.g., provider order templates), and continuous provider training. Altogether, these system adoptions will enable an annual reach of >19,000 patients across the healthcare system for facilitating cancer risk assessment and access to risk-based cancer healthcare (e.g., screenings, clinical trials). Other I-CHER projects with clinical components are still undergoing active testing and will soon be considered for possible system-wide sustainability, including (1) utilization of a training video for dental providers to help them formulate their own approach for effective and culturally appropriate communications with black men around oropharyngeal cancer, and (2) development of a provider educational curriculum tool to aid appropriate decision making around colorectal cancer screening recommendations (i.e., CSDGs; Table 2).

#### 2.3.3. Research-Driven Advocacy for State Policy

Supported by the AC and UICC COE leadership, I-CHER investigators have directly contributed to two state laws focused on cancer genetic services and clinical trial infrastructure, which were sponsored by CAB members. To facilitate the translation of I-CHER research to policy, the AC leveraged existing COE structure for research-driven policy advocacy, including (1) engagement of CAB members who were advocates (ACS, ACS CAN) and legislative champions (state representatives); and (2) COE team members’ partnerships with state health department (e.g., evaluator and technical assistance for Illinois State’s cancer control programs) [77,78]. Annually, the AC learned about emerging policy goals from CAB members, prior to the beginning of the legislative session, during which CAB members invited I-CHER study PIs to share scientific testimonies. The AC and UICC COE also facilitated constituent education about emerging policies, including interviews with I-CHER study PIs and CAB members. Finally, emerging policies were adopted as priorities during yearly UICC Advocacy Days, led by the UICC Director and I-CHER PI.

A challenge concerning leveraging I-CHER research for state laws was understanding which strategy to promote when, based on priorities set by legislative champions and advocates. Specifically, dissemination meetings were helpful for building awareness about legislative champions (e.g., state representatives) and policy advocates (e.g., ACS, ACS CAN) on the CAB; however, dissemination did not necessarily always result in policy development. To monitor policy interests and agenda setting, COE members on the AC regularly attended partners’ advocacy and legislative update meetings, especially before and during legislative sessions. After identifying policies that could benefit from I-CHER research, the COE-fueled AC equipped study teams with policy development, in line with their relevant I-CHER research findings, while emphasizing common issues impacting legislation (e.g., long-term cost savings).

In part due to I-CHER CAB leaders and researchers, Illinois Public Act 103-0914 mandates coverage for genetic testing and subsequent risk-based cancer screenings, with a cost cap of $50, allowing for greater uptake of clinical-level workflows tested by I-CHER research through a reduction in financial barriers to genetic testing. Relevant I-CHER findings from two RSGs (TestMiGenes, LaLiSa) emphasized the importance of coverage, given that research innovations were possible through institutional coverage of costs for genetic services. Similarly, the FOR ME study (Table 2) demonstrated the value of community involvement in clinical trial education and recruitment strategies and contributed to foundational evidence that directly supported adaptations to the Cancer Clinical Trial Participation Program Act, which scaled I-CHER’s focus on community involvement by addressing the ancillary costs and infrastructure gaps that frequently prevent MUA/P and other low-income individuals from enrolling in life-saving trial studies.

## 3. Discussion

The I-CHER Center is a significant case study in cancer health equity efforts, with a focus on how to lead large-scale research initiatives in precision cancer health equity. The operational evolution of the Center is anchored in a continuous feedback loop between anticipated implementation risks and iterative structural solutions, as summarized in Table 3 (below). The challenges and risks encountered during the implementation of the Center’s essential functions and operations, along with the lessons learned from the corrective solutions imposed, can provide a practical roadmap for under-resourced academic and healthcare systems that are seeking to bridge the gap between precision cancer health and health equity. This framework highlights how initial ‘missteps’—ranging from regulatory friction across a three-board review triad to the challenges of integrating research into high-pressure clinical workflows—were adequately addressed through deliberate, solution-based design to not only ensure the feasibility of individual studies, but also generate scalable impact.

### 3.1. The I-CHER Model: Insights from Structural Evolution and Lessons in Implementation

Beyond implementation challenges and solutions, our case report offers three major insights into launching large-scale cancer health equity centers within under-resourced settings.

First, our case report highlights the value of implementing equity-driven research centers within the safety net ecosystems that serve MUA/P residents and low-income populations at large. Equipping clinicians, faculty, and leaders with the resources to test innovations on top of the culturally appropriate and accessible care they provide unique opportunities to ensure scientific solutions can reach all populations and to test their efficacy among communities with multilevel health risks. By elevating the safety net workforce in research—as PIs, co-investigators, and key personnel—we naturally foster a sense of ownership in novel scientific solutions that can facilitate implementation and expedite efficacy testing. Our case report further highlights a robust institutional capacity for clinical capacity to provide precision cancer health, which was further grown through the I-CHER Center. The case of UI Health and Mile Square should be used to consider how quality improvement and other clinical service interventions can open doors for methodological innovations (e.g., “high tech”—artificial intelligence/machine learning; “high touch”—decentralized resource navigation). In turn, if successful, these interventions may be highly likely to be adopted and sustained in practice.

Second, our case report highlights the utility of administrative components required for NCI designation and demonstrates the feasibility of emerging cancer centers to implement administrative components. This is particularly important regarding COE, which is a crucial component for ensuring cancer research is responsive to evolving community needs and is effectively translated to practice and policy. Our case report further highlights how COE infrastructure may be particularly impactful when operated within accessible academic settings like MSIs and with internal linkages to the public safety net ecosystem for communities most at risk of cancer.

Finally, our case report highlights the importance of integrating executive and institutional leadership in large-scale research initiatives focused on cancer equity. The I-CHER Center was able to address resource constraints and accomplish multilevel buy-in because the Center PI was the UICC Director. In line with other research centers, COE and clinical leaders with relevant expertise actualized the mission and operations of the I-CHER Center. Having the UICC Director as the PI significantly increased how resources were allocated and shared between the research center and its institution. There are further multilevel benefits to having cancer center directors directly incorporated in cancer health equity centers, including more opportunities to engage with community and clinical leaders, and greater exposure to the value and impacts of COE and multisectoral partnerships across different scientific disciplines.

### 3.2. Limitations and Future Directions

Despite its early successes, I-CHER’s model for a cancer center rooted in achieving equity in precision cancer health is nonetheless subject to limitations. First, while the I-CHER Center offers a model for an equity- and mission-driven administrative infrastructure, it may not universally serve as a suitable or immediately replicable blueprint for Minority Serving Institutions (MSIs) or FQHCs with emerging research capacity or those without a demonstrated history of engaging in institutional research. Indeed, the I-CHER Center leveraged an already established, robust infrastructure for internal FQHC-academic partnerships and for community-academic partnerships. Given this track record, the I-CHER Center leveraged institutional resources and capacity (e.g., cost-sharing) that may not be immediately possible for other MSIs and FQHC/safety net systems. A related limitation is the potential challenge to generalizability; while I-CHER’s deep partnership with MSHC is a strength, the unique local context of this academic-FQHC partnership and proximity to medically underserved communities within the city of Chicago, IL, may limit the direct transferability of certain elements of I-CHER’s model to other centers without local proximity and significant adaptation. Another limitation is that the selection of the Center’s initial research portfolio did not evaluate and prioritize projects in terms of how they addressed fundamental theoretical uncertainties pertaining to the multilevel drivers and multi-faceted context of precision cancer health. This is a key criterion for future research centers, as they consider study selection, to maximize research impacts and advance our collective knowledge of precision cancer health.

## 4. Conclusions

I-CHER Center provides a reproducible blueprint for integrating multi-level partnerships, diverse research, and proactive policy advocacy to achieve health equity in the context of precision cancer medicine. By simultaneously focusing on the molecular basis of disparities, the systems of care, and the policies by which they are governed, this model streamlines the delivery of holistic solutions where they are needed most.

## Figures and Tables

**Table 1:** Aims and activities of the I-CHER Center components.

Component	Aims	Activities
Administration Core (AC)	(1) Provide overall support through accessible standardized operating procedures, norms, and programming practices. (2) Facilitate multi-sectoral engagement, dissemination, and translation of I-CHER research into practice and policy.	Tracking and assurance of fiscal, regulatory, and programming for compliance. Oversee communications, meetings, coordination, and alignment with the I-CHER Center’s standardized operation. Oversight of research translation, including CAB meetings, meetings with decision makers, etc.
Investigator Development Core (IDC)	(1) Grow a pipeline of interdisciplinary Early Career Faculty focused on cancer health equity research. (2) Provide structured career development opportunities to Project PIs, Postdoctoral fellows and pilot awardees.	Structured career and leadership development opportunities in community-engaged cancer research. Pilot project program for incubating cancer disparities research.
Community Advisory Board (CAB)	(1) Serve as liaison between the I-CHER Center and the communities most impacted by cancer in the region, state, and nationally. (2) Provide guidance to the research subawards to enhance the appropriateness and effectiveness of their outreach efforts, recruitment processes, data collection procedures, intervention delivery, and dissemination of their research findings and products. (3) Policy goal—Inform policy initiatives and advocacy efforts that are responsive to communities’ needs. (4) Inform dissemination and sustainability plans of each of the subawards.	Connect subaward PIs, I-CHER leaders, and pilot awardees to community members and leaders and help promote community engagement. Provide strategic direction in partnership development by identifying areas of opportunity Provide 1:1 feedback and guidance on subaward projects. Identify and undertake planning for participating in advocacy forums and policy initiatives, e.g., coordinate with ACS on the ACS advocacy days, and the UICC Office of Community Engagement and Health Equity (CEHE) will help coordinate efforts. Co-implement dissemination strategies (e.g., CEHE’s annual report, website material, townhalls, webinars, local radio shows; national and local podcasts; local health fairs—e.g., Fiesta del Sol, Black Women’s Expo) to promote community awareness of study findings on multi-level solutions for cancer equity and next steps in research, practice, and policy.

**Table 2:** Summary of I-CHER research activities, projects, and teams.

Center Activities and Projects			
Research Scholar Grants (RSGs)			
Title	Aims	Study Design	Research Team
Developing and Optimizing Best Practices for Population-Based Cancer Genetic Services in FQHCs (TestMiGenes Study) *	(1) Compare approaches for cancer genetic services delivery among FQHC patients. (2) Evaluate the implementation of approaches.	Comparative effectiveness trial, using a hybrid implementation-effectiveness design, to examine two approaches (genetic testing by PCP vs. referral to a genetic counselor) to enhance access to cancer genetic risk assessment in Federally Qualified Health Centers (FQHCs).	FQHC & UICC PI FQHC & UICC Co-Is FQHC Navigation & Genetic Services Teams
Fostering Opportunities in Research through Messaging and Education (FOR ME Study) *	(1) Identify barriers to participation for precision cancer oncology and other clinical trials for Black breast cancer patients. (2) Create and pilot-test a narrative video intervention in safety net settings.	Qualitative data collection and video tool evaluation by patients, providers, and community advocates to develop a narrative intervention that promotes inclusive clinical trial participation.	Oncology & UICC PI Safety Net Oncology & UICC Co-Is Community Consultants
Latinas Lideres en Salud (LaLiSa) Study	(1) Compare intervention effects on Latinas due for genetic testing. (2) Compare intervention effects on network members. (3) Identify contributing neighborhood and network factors.	Comparative effectiveness RCT for two interventions (train-the-trainer/empowerment vs. education) aimed at improving access to cancer genetic services by addressing multilevel risk factors and social networks.	FQHC & UICC PI Community Co-I FQHC Co-Is
**Clinical Scientist Development Grants (CSDGs)**			
**Title**	**Aims**	**Study Design**	**Research Team**
Optimizing colorectal cancer risk assessment for targeted screening using family history in underserved populations *	(1) Investigate gaps in cancer-related family history documentation. (2) Develop a tool to optimize family history collection and documentation. (3) Pilot test a provider-tailored intervention.	Mixed methods/pilot intervention study focused on reducing disparities in risk assessment for colorectal cancer via medical chart review, systematic review, and pilot trial protocol.	Clinician & UICC PI FQHC & Primary Care Mentors
Communication tool development for Black oral and pharyngeal cancer (OPC) patients *	(1) Adapt an existing OPC patient communication tool for identifying OPC risks. (2) Adapt a culturally specific communication tool. (3) Pilot test tool in community-based dental clinics.	Community-based participatory research (CBPR)-grounded mixed-methods implementation study with focus groups, surveys, simulation, and pilot testing components, focusing on Black men, aimed to develop culturally specific tools that ensure equitable access to communication regarding OPC.	Clinician & UICC PI Community Consultants
**Postdoctoral Fellowship (PF)**			
**Title**	**Aims**	**Study Design**	**Research Team**
Racial ethnic variation in ACKR1 *	(1) Determine endothelial ACKR1 role and prognostic potential in immune infiltration of triple-negative breast cancer. (2) Define the differential effects of ribociclib on neutropenia in a mouse model and the impact of Duffy null serotype on CDK4/6 inhibitor treatment regions.	Translational genomic cohort study with biomarker analysis using TCGA and patient cohort data to investigate molecular determinants of precision cancer equity (e.g., genetic risk by ancestry), including genetic variants [ACKR1] and their association with immune response.	UICC Trainee UICC Mentors (Deputy Director, AD of COE)
**Pilot Projects**			
**Title**	**Aims**	**Study Design**	**Research Team**
Data-driven assessment of cervical cancer screening guideline concordance *	(1) Determine cervical cancer screening rates and multilevel contributing risks in Chicago. (2) Determine the rate of adequate prior screening in women over 66 years old.	Retrospective cohort/EHR data analysis study utilizing a large research consortium (CAPriCORN network) to identify system-level and multilevel risk factors contributing to cervical cancer screening disparities.	Clinician & UICC PI UICC Mentors (AD of COE, Safety Net Co-Is)
Prevention and Screening of Cancer in the Emergency Department (ED) *	(1) Assess cancer screening rates in the ED setting. (2) Implement an EMR algorithm to identify patients overdue for screening. (3) Identify multilevel barriers to screening.	EHR-based mixed methods implementation study focused on improving awareness of cancer screening for emergency department patients via health informatics tools.	ED Faculty & UICC PI UICC Mentors (AD of COE)
Surface-Engineered NK T/Trastuzumab cells targeted immunotherapy *	(1) Evaluate the effect of surface-engineered NK/Trastuzumab cells (SE-NK/Tz cells) on human HER2-low expressed by 4T1 cells in vitro.	In vivo and in vitro pre-clinical/basic science study.	Clinician & UICC PI UICC Mentor
Using AI/ML to mitigate racial disparities in early detection of lung cancer *	(1) Pilot clinical testing of circulating fragmentomics in LDCT patients. (2) Assess racial disparities using multimodal testing.	Prospective clinical testing/AI-driven feasibility study focused on equitable access to early lung cancer detection through primary care/FQHC-accessible screening tools.	Clinician & UICC PI FQHC Co-Investigators (Mile Square CEO, Associate Chief Medical Officer)
Analyzing non-genetic mechanisms of resistance to KRAS-specific therapies to improve drug response	(1) RNAseq and methylation array of KRAS G12D resistant models. (2) Identify drug sensitivity biomarkers and vulnerability targets. (3) Validation of biologically relevant drug-resistant biomarkers and alternative therapeutic approaches.	In vitro pre-clinical/basic science study.	UICC PI & Mentor
Food is Medicine: A Lifestyle Modification for Black Breast Cancer Survivors with Cardiovascular Risk	(1) Adapt an existing Food is Medicine (FIM) intervention for Black BCS (2) Pilot test six sessions of the FIM + BCS intervention (3) Assess the intervention’s acceptability and feasibility.	Prospective FIM intervention for breast cancer survivors.	Clinician & UICC PI I-CHER CAB members as Community Consultants

* Abbreviated from full study/project name.

**Table 3:** Summary of I-CHER implementation risks/challenges and strategic solutions.

Essential Function	Implementation Risk(s)/ Challenge(s)	Solution(s)
Research Project Selection	Review Logic Mismatch: Determining whether community or academic reviews should lead selection discussions	Sequential Review Processes: Operationalized a “rigor-first” academic review followed by a community “fit” review, mirroring national standards
	Gaps in Scope: Initial applications lacked focus on cancer survivorship and direct intervention testing	Strategic Portfolio Refinement: Created new criteria attracting survivorship projects and expanded pilot timelines to two years
	Evaluator Preparations: Need for better training on the Center’s priorities and structured evaluation tools	Formal Reviewer Trainings: Implemented mandatory training and standardized, structured scoring templates
Monitoring Selected Research Projects	Regulatory Triad Friction: Substantial delays expected from navigating three distinct review committees (FQHC, Cancer Center, and UIC)	Integrated Regulatory Support: Incorporated senior regulatory specialists directly into Center operations and mandatory awardee consultations
	PI Bandwidth: Early-career investigators faced high clinical and teaching loads, limiting their capacity for complex regulatory monitoring	Operational Synchronization: Formalized monthly leadership meetings and focused “research consultations” with FQHC operations teams
	Operational Realities: Anticipated and experienced delays in recruitment and primary data collection	Strategic Flexibility: Granted no-cost extensions and encouraged EMR data abstraction to mitigate recruitment hurdles
Translation into Practice	System Integration: Numerous challenges in implementing research interventions within high-pressure FQHC clinical workflows	Operational Leveraging: Reframed research projects as Quality Improvement (QI) initiatives to align with mandatory FQHC reporting
	Stakeholder Burden: High-level clinical leaders lacked time for traditional dissemination meetings	Embedded Education: Utilized “High Tech” solutions, including EMR-based After Visit Summaries (AVS) to automate screening notifications
Translation into Policy	Relevance Gap: Difficulties identifying specific scientific findings that were ready for or relevant to state-level policy	Strategic Partnerships: Coordination from the Associate Director of COE that linked study teams with the ACS Cancer Action Network
	Advocacy Disconnect: Absence of a structured pathway connecting academic researchers with policy advocacy groups	Legislative Testimony: Facilitated researcher presentations to state-level decision makers, which contributed to the passage of two state laws

## Data Availability

Please contact ymolin2@uic.edu for interest in access and use of I-CHER Center data.

## Abbreviations

The following abbreviations are used in this manuscript:

UIC	University of Illinois Chicago
I-CHER	Illinois Cancer Health Equity Research (Center)
UICC	University of Illinois Cancer Center
COE	(Office of) Community Outreach and Engagement
ACS	American Cancer Society
CHERC	Cancer Health Equity Research Center (program)
CAB	Community Advisory Board
CRTEC	(Office of) Cancer Research Training and Education Coordination
UI Health	University of Illinois Health (System)
PI	Principal Investigator
Admin core	Administrative Core
IDC	Investigator Development Core
ChicagoCHEC	Chicago Cancer Health Equity Collaborative
CEHE	(Office of) Community Engagement and Health Equity
Co-PI(s)	Co-Principal Investigator(s)
Co-I(s)	Co-Investigator(s)
CMO	Chief Medical Officer
GRA	Graduate Research Assistant
RCT	Randomized Controlled Trial
CBPR	Community-Based Participatory Research
OPC	Oral and Pharyngeal Cancer
FIM	Food Is Medicine
RSG	Research Scholar Grant
BCS	Breast Cancer Survivors
CSDG	Clinical Scientist Development Grant
PF	Postdoctoral Fellowship
CAPriCORN	Chicago Area Patient-Centered Outcomes Research Network
TCGA	The Cancer Genome Atlas
EHR	Electronic Health Record
ED	Emergency Department
AI/ML	Artificial Intelligence/Machine Learning
SDoH	Social Determinants of Health
COVID-19	Coronavirus Disease
QI	Quality Improvement
MSHC	Mile Square Health Center
FQHC	Federally Qualified Health Center
MSI	Minority Serving Institution
UDS	Uniform Data System
AANAPIS	Asian American and Native American Pacific Islander Serving
ACS CAN	American Cancer Society Cancer Action Network
